# PPAR**δ** Agonist GW501516 Inhibits PDGF-Stimulated Pulmonary Arterial Smooth Muscle Cell Function Related to Pathological Vascular Remodeling

**DOI:** 10.1155/2013/903947

**Published:** 2013-03-27

**Authors:** Guangjie Liu, Xuan Li, Yan Li, Xin Tang, Jie Xu, Ran Li, Peng Hao, Yongchang Sun

**Affiliations:** ^1^Department of Respiratory Medicine, Beijing Tongren Hospital, Capital Medical University, Beijing 100730, China; ^2^Nankai University Eye Hospital, Tianjin 300020, China; ^3^Tianjin Eye Hospital, Tianjin Key Laboratory of Ophthalmology and Visual Science, Clinical College of Ophthalmology, Tianjin Medical University, Tianjin 300020, China

## Abstract

Pulmonary arterial hypertension (PAH) is a severe and progressive disease, a key feature of which is pulmonary vascular remodeling. Growth factors, cytokines, and lipid mediators are involved in this remodeling process. Recent reports suggest that the peroxisome proliferator-activated receptors (PPARs) play important roles in the regulation of cell growth and differentiation as well as tissue wounding and repair. In this study, we examined the role of PPAR**δ** in the regulation of proliferation, migration, collagen synthesis, and chemokine production in human pulmonary arterial smooth muscle cells (HPASMCs). The data showed that PPAR**δ** was the most abundant isoform in HPASMCs. PPAR**δ** was upregulated in HPASMCs treated with PDGF, which is the major mediator in pulmonary vascular remodeling. Activation of PPAR**δ** by GW501516, a specific PPAR**δ** ligand, significantly inhibited PDGF-induced proliferation in HPASMCs. The inhibitory effect of GW501516 on HPASMCs was associated with decreased expression of cyclin D1, cyclin D3, CDK2, and CDK4 as well as increased expression of the cell cycle inhibitory genes G0S2 and P27^kip1^. Pretreatment of HPASMCs with GW501516 significantly inhibited PDGF-induced cell migration and collagen synthesis. GW501516 also significantly attenuated TNF-mediated expression of MCP-1. These results suggest that PPAR**δ** may be a potential therapeutic target against the progression of vascular remodeling in PAH.

## 1. Introduction

Pulmonary arterial hypertension (PAH) is a life-threatening disease characterized by increased pulmonary vascular resistance and pulmonary arterial pressure leading to right heart failure. The etiology and pathogenesis of PAH are complex and incompletely understood. Pulmonary vascular remodeling is a hallmark of most forms of PAH, including both primary and secondary PAHs. Accumulation of extracellular matrix including collagen as well as vascular smooth muscle cell proliferation and migration contribute to the muscularization of the pulmonary arterial wall, leading to a severe decrease of the cross-sectional area and therefore an increase in the right ventricular afterload [1, 2]. Growth factors and cytokines participate in the processes of abnormal vascular remodeling, inflammation, and cell proliferation involved in PAH [3]. PDGF is a potent mitogen involved in cell proliferation and migration. Active PDGF is composed of polypeptides (A and B chains) that form homo- or heterodimers that stimulate its cell surface receptors. Studies show that PDGF-B and the PDGFRb are primarily required for the development of the vasculature. PDGF is synthesized by many different cell types including vascular smooth muscle cells (VSMCs), vascular endothelial cells (ECs), and macrophages. PDGF induces the proliferation and migration of VSMCs and has been proposed to be a key mediator in the progression of several fibroproliferative disorders, such as atherosclerosis, lung fibrosis, and PAH [4, 5]. Inflammation has a key role during the development of PAH. Levels of cytokines and chemokines are elevated in the blood of patients with PAH (e.g., TNF*α*, soluble TNF-receptor type I, interleukin 1, and MCP-1) [6]. It is clear that PAH has a multifactorial pathobiology, and the current understanding of the mechanisms of PAH has facilitated the development of novel therapeutic strategies for PAH focused on the cell proliferation, vascular remodeling, and inflammation involved in PAH. 

Peroxisome proliferator-activated receptors (PPARs) are ligand-activated transcription factors and members of the nuclear hormone receptor superfamily that includes three isoforms: PPAR*α*, PPAR*γ*, and PPAR*β*/*δ*. Ligand-activated PPARs form heterodimers with a retinoid X receptor (RXR) and bind to specific PPAR responsive elements (PPREs) to regulate target gene expression. The activation of PPARs modulates numerous biological processes including energy homeostasis, cell proliferation and differentiation, fatty acid catabolism, and adipogenesis. Although most studies have shown that PPAR*α* and PPAR*γ* exert anti-inflammatory, antiproliferative, and antiangiogenic properties in cardiovascular cells, the role of PPAR*δ* in vascular pathophysiology is poorly understood [7, 8]. Intriguingly, recent literature suggests that the ligand activation of PPAR*δ* induces the terminal differentiation of keratinocytes and inhibits cell proliferation [9, 10]. Prostacyclin (PGI2), the predominant prostanoid released by vascular cells, is a putative endogenous agonist for PPAR*δ*, and the administration of PGI2 or its analogues represents a significant advance in PAH therapy [11, 12]. Overexpression of PGI2 synthase inhibits the growth of VSMCs and prevents neointimal formation in rat carotid arteries after balloon injury [13, 14]. In addition, inflammation plays a significant role in altering pulmonary vascular function during the development of PAH. The plexiform lesions that characterize severe PAH are surrounded by macrophages, T and B lymphocytes, and dendritic cells [15, 16]. Therefore, agents that target the generation of the inflammatory stimuli in the pulmonary vascular wall may reduce vascular dysfunction and attenuate the development or progression of PAH. Increasing evidence indicates the anti-inflammatory properties of PPAR*δ* activation in some cell types and animal models. PPAR*δ* activation inhibited the induction of MCP-1 and intercellular adhesion molecule-1 (ICAM-1) genes in a cardiac ischemia/reperfusion model [17]. Together, these observations raise the possibility that PPAR*δ* mediates vascular remodeling by mitigating vascular smooth cell proliferation, extracellular matrix (ECM) production, and inflammation.

In the present study, we aimed to define the functional significance of PPAR*δ* in pulmonary arterial smooth muscle cells. According to our data, PPAR*δ* is abundantly expressed in HPASMCs, and we demonstrate that PDGF stimulation increases PPAR*δ* expression by 2- to 3-fold in HPASMCs. Activation of PPAR*δ* by GW501516 inhibits the PDGF-induced proliferation and migration of HPASMCs as well as collagen synthesis. Moreover, GW501516 exerts its inhibitory effects by regulating the PDGF-induced expression of cell cycle regulatory genes and attenuates the TNF*α*-induced MCP-1 expression in HPASMCs.

## 2. Materials and Methods

### 2.1. Materials

GW501516 was purchased from Calbiochem (San Diego, CA, USA). Platelet-derived growth factor- (PDGF-) BB and tumor necrosis factor- (TNF-) *α* were purchased from R&D (Minneapolis, MN, USA). Antibodies against PPAR*δ* (sc-74440) or actin (sc-1616) were purchased from Santa Cruz Biotechnology (Santa Cruz, CA, USA). 

### 2.2. Cell Culture

The human pulmonary arterial smooth muscle cells (HPASMCs) and human pulmonary arterial endothelial cells (HPAECs) were purchased from Lonza. HPASMCs and HPAECs were cultured according to the supplier's instructions. Cells of passage 4–7 were subjected to serum starvation for 24 hours before being used for the experiments.

### 2.3. BrdU Incorporation Assay

Cellular proliferation was assayed with a kit from Roche that monitors the incorporation of BrdU into newly synthesized DNA. BrdU was detected using an anti-BrdU-peroxidase conjugate in accordance with the manufacturer's instructions. The amount of BrdU incorporated was determined by measuring the absorbance at 450 nm. 

### 2.4. Cell Migration: Transwell Assay

Migration assays were performed using a Boyden chamber. HPASMCs were digested with 0.05% trypsin and dispersed into homogeneous cell suspensions that were placed on the upper surface of an 8 *μ*m pore size chamber. The upper chamber contained medium with GW501516 (3 *μ*M), and cellular migration was induced by adding PDGF-BB (10 ng/mL) to the lower chamber. After 16 h, the nonmigrating cells were removed, and the membrane was fixed and stained. The results are expressed as the number of migrated cells per square millimeter. 

### 2.5. Western Blot Analysis

Cell lysates were prepared with RIPA buffer containing the complete protease mix (Roche). Fifty micrograms of protein was subjected to SDS-PAGE electrophoresis and electrotransferred to PVDF membrane. Membranes were processed as described by the manufacturer of the antibodies. The immunoreactive bands were detected by chemiluminescence (Millipore) and quantified by densitometry. 

### 2.6. ^3^H-Proline Incorporation Assay

Serum-starved HPASMCs were incubated with 1 *μ*Ci/mL ^3^H-proline (Amersham) along with PDGF after pretreatment with or without GW501516. After 24 h, the cells were washed with ice-cold PBS, treated with ice-cold 15% trichloroacetic acid (TCA) for 1 hour and then washed with ice-cold ethanol. The precipitants were solubilized in lysis buffer (2% SDS, 1 mM EDTA and 40 mM Tris, pH 7.4) for the liquid scintillation counting assay. The radioactive counts per minute (CPM) representing the amount of newly synthesized collagen were normalized to the total protein content. 

### 2.7. Real-Time Reverse Transcription PCR

Total RNA was extracted using TRIzol (Invitrogen, Carlsbad, CA, USA). The cDNA was generated using 2.5 *μ*g of total RNA with the MultiScribe Reverse Transcriptase Kit (Applied Biosystems, Foster City, CA, USA). Real-time PCR reactions were performed using a Bio-Rad iCycler with the SYBR Green Supermix (Bio-Rad), according to the manufacturer's protocol. The primer sequences were as follows: GAPDH: forward, 5′-agggctgcttttaactctggt-3′, reverse, 5′-ccccacttgattttggaggga-3′; PPAR*α*: forward, 5′-cttcaacatgaacaaggtcaaagc-3′, reverse, 5′-agccatacacagtgtctccatatca-3′; PPAR*δ*: forward, 5′-gggcatgtcacacaacgctat-3′, reverse, 5′-gcattgtagatgtgcttggagaa-3′; PPAR*γ*: forward, 5′-tctctccgtaatggaagacc-3′, reverse, 5′-gcattatgagacatccccac-3′; CyclinD1: forward, 5′gagaccatccccctgacggc-3′, reverse, 5′-tcttcctcctcctcggcggc-3′; CyclinD3: forward, 5′-attctgcaccggctctctc-3′, reverse, 5′-gcttcgatctgctcctgaca-3′; CDK2: forward, 5′-gctttctgccattctcatcg-3′, reverse, 5′-gtccccagagtccgaaagat-3′; CDK4: forward, 5′-atgttgtccggctgatgga-3′, reverse, 5′-caccagggttaccttgatctcc-3′; G0S2: forward, 5′-aaagatataagcggcccccg-3′, reverse, 5′-ggaggcgggaatgaccttag-3′; P27kip1: forward, 5′-tgcaaccgacgattcttctactcaa-3′, reverse, 5′-caagcagtgatgtatctgataaacaagga-3′; MCP1: forward, 5′-ccccagtcacctgctgttat-3′, reverse, 5′-agatctccttggccacaatg-3′. The results were stated as the fold difference expression for each gene compared to that of GAPDH, using the 2^−ΔΔCt^ method. 

### 2.8. Statistics

All measurements were expressed as mean ± SD. Student's *t*-test was used for comparison between two groups, whereas an analysis of variance (ANOVA) was performed for multiple comparisons. All statistical analyses were performed using SPSS (v13.0). Statistical significance was defined as *P* < 0.05.

## 3. Results

### 3.1. PPAR Isoforms in HPASMCs and HPAECs

**Figure 1:**
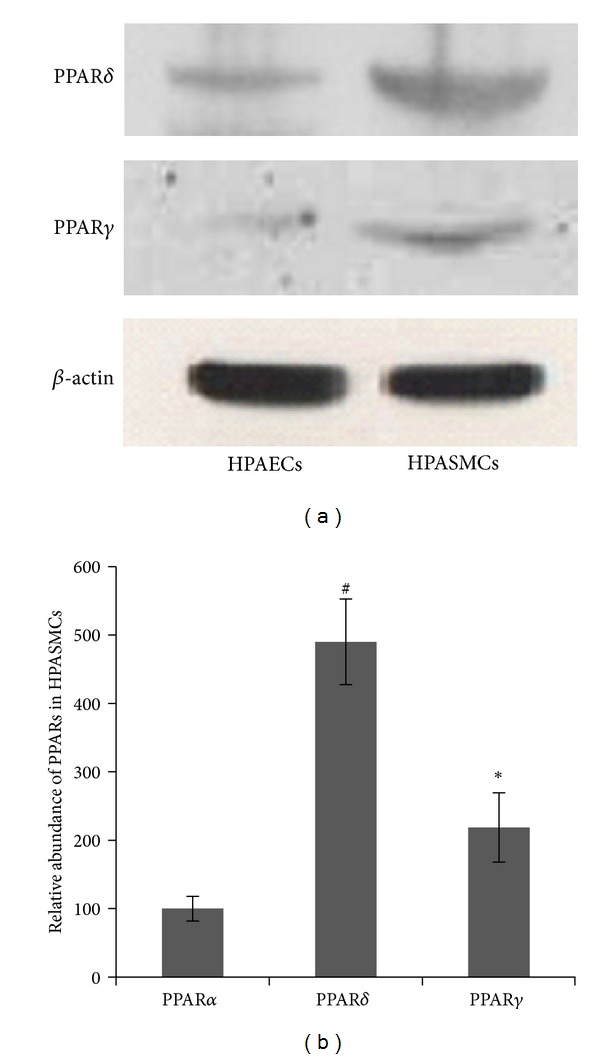
Expression patterns of PPAR isoforms in human pulmonary vascular cells. (a) Expression of PPAR*δ* or PPAR*γ* is higher in HPASMCs compared with HPAECs. Results were similar in three independent experiments. (b) PPAR*α* mRNA level in HPASMCs was set to 100.0. Other mRNA levels were expressed relative to this value. Experiments were performed in triplicate and repeated a minimum of three times. ^#^ *P* < 0.01, *P < 0.05 versus PPAR*α*.

Using western blot analysis, we demonstrated that PPAR*δ* protein was expressed in both cultured HPASMCs and HPAECs; moreover, expression of PPAR*δ* was higher in HPASMCs than in HPAECs. Compared with PPAR*γ*, a relatively high level of PPAR*δ* protein was observed in both HPASMCs and HPAECs (Figure 1(a)). Real-time quantitative PCR confirmed the presence of the three PPAR isoforms in HPASMCs. The relative abundance for PPAR*α*, PPAR*δ*, and PPAR*γ* mRNA was 1.00 : 4.90 : 2.19 (Figure 1(b)). These data document the differential expression patterns of the PPAR isoforms present in cultured HPASMCs. 

### 3.2. PDGF Induced Expression of PPAR*δ* in a Dose- and Time-Dependent Manner in HPASMCs

**Figure 2:**
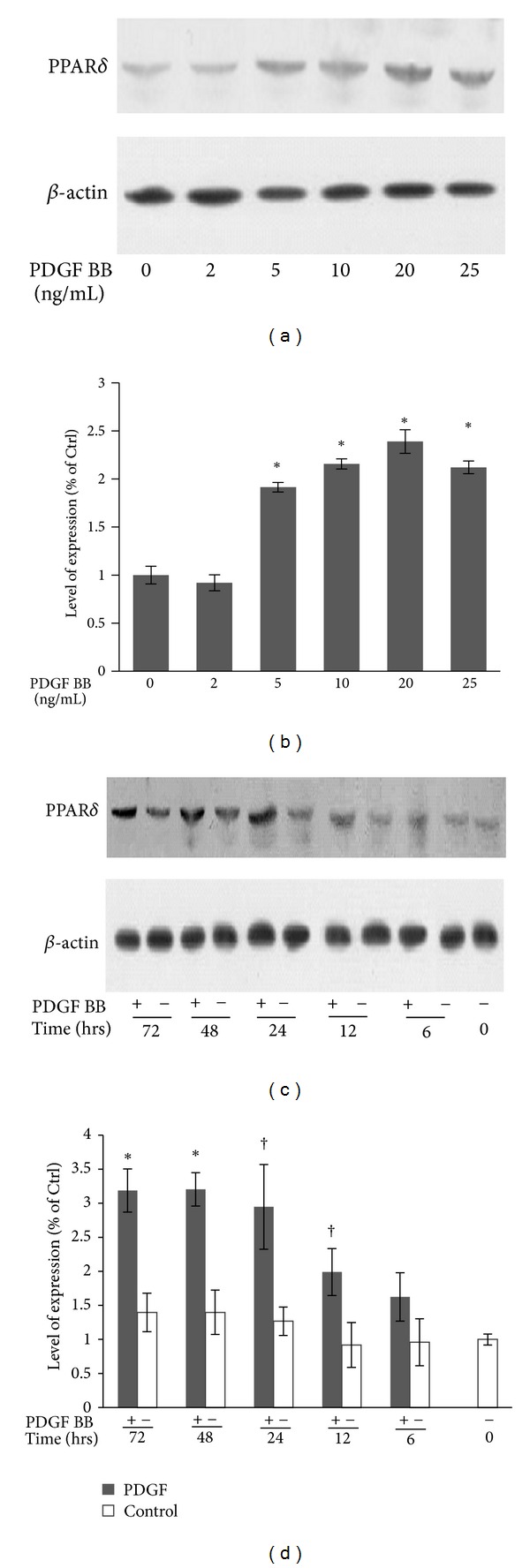
PDGF upregulates PPAR*δ* expression in a time- and dose-dependent manner. HPASMCs were made quiescent for 24 h and then treated with PDGF with increasing concentrations of PDGF for 24 h to study the effect of dose (a, b) or treated with PDGF (10 ng/mL) to study the effect of time, as indicated (c, d). The results were quantified by densitometry and normalized with respect to *β*-action; the control was set to 1.0. Results are expressed as the means ± SD of at least three independent experiments. *P < 0.01 versus control nontreated cells; ^†^ *P* < 0.05 versus control nontreated cells.

PPAR*δ* has been linked to proliferation in some cell lines; furthermore, it was recently reported that PPAR*δ* is upregulated during vascular lesion formation [18]. Whether PPAR*δ* is associated with pulmonary vascular cells is unclear. Based on the investigation of abundant PPAR*δ* in HPASMCs described above, we tested the expression of PPAR*δ* in HPASMCs treated with PDGF, a key mediator in PAH pathogenesis and vascular remodeling. HPASMCs were treated with different doses of PDGF for 24 h. The dose response of PDGF-induced PPAR*δ* production was determined. As shown in Figure 2(a), western blot analysis of the cell lysates indicated that PDGF upregulated PPAR*δ* protein expression in a dose-dependent manner. A significant increase was observed at a PDGF concentration as low as 5 ng/mL, whereas the maximal increase was obtained at a concentration of 20 ng/mL. We tested the time course of the PDGF-induced PPAR*δ* production in HPASMCs. HPASMCs were treated with 10 ng/mL PDGF for 0, 6, 12, 24, 48, or 72 h. As shown in Figure 2(c), western blot analysis revealed that PDGF upregulated PPAR*δ* protein expression in the cell lysates as early as 6 h and reached a plateau at 24 h. These results demonstrate that PDGF induces PPAR*δ* protein expression in a dose- and time-dependent manner in HPASMCs.

Next, we investigated the effects of PPAR*δ* on cell biological processes of HPASMCs, including proliferation, migration, collagen synthesis, and chemokine production.

### 3.3. Ligand Activation of PPAR*δ* Inhibited PDGF-Induced Proliferation of HPASMCs

**Figure 3:**
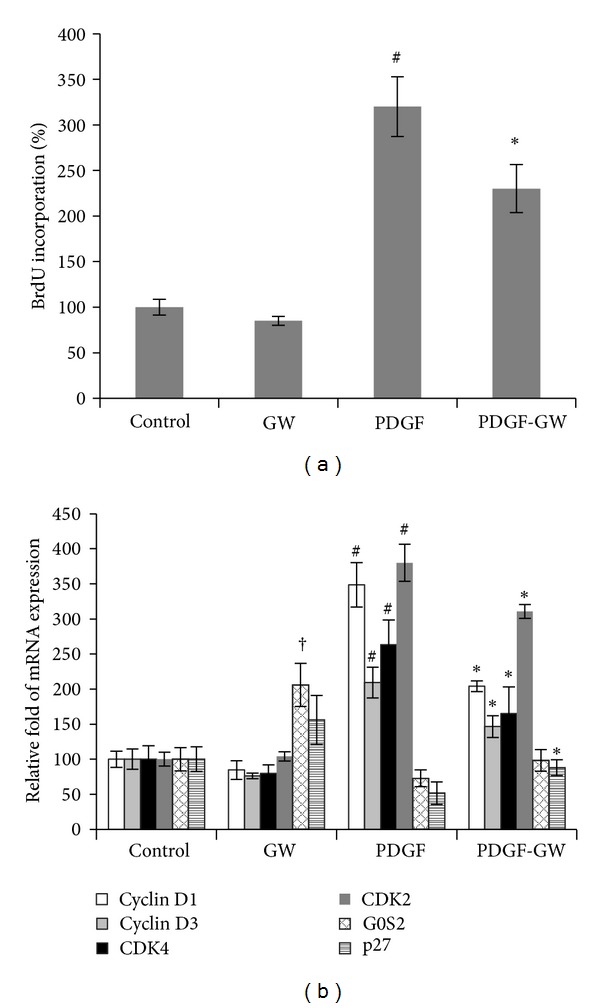
Effects of GW501516 on cell proliferation and expression of multiple cell cycle regulatory genes in HPASMCs. (a) HAPSMCs were pretreated with GW501516 (GW, 3 *μ*M) for 6 h prior to 36 h of PDGF-BB treatment (PDGF, 10 ng/mL). DNA synthesis was measured by BrdU incorporation assay. (b) mRNA levels of multiple cell cycle regulatory genes were determined in HPASMCs. Experiments were performed in triplicate and repeated a minimum of three times. ^#^ *P* < 0.01, ^†^ *P* < 0.05 versus control; *P < 0.05  versus PDGF-treated cells.

We studied the effect of GW501515, a highly selective PPAR*δ* agonist, on the proliferation of HPASMCs. In HPASMCs, BrdU incorporation was slightly reduced following GW501515 treatment. The HPASMCs were pretreated with GW501515 for 6 h followed by PDGF induction. BrdU incorporation was significantly reduced, by ~28%, after GW501516 pretreatment (Figure 3(a)). Our data indicated that the ligand activation of the PPAR*δ* decreased PDGF-induced proliferation of HPASMCs. 

The effect of GW501516 was examined when serum was used to replace PDGF. Inhibition of cell proliferation was also observed in HPASMCs in response to GW501516 in culture medium containing FBS (data not shown).

To explore the potential mechanisms by which PPAR*δ* influences the proliferation of HPASMCs, the mRNA levels of cell cycle regulatory genes, including cyclins and the cyclin-dependent kinases (CDKs), and cell cycle inhibitory genes were examined. As shown in Figure 3(b), PDGF induced significant increases in the mRNA levels of cyclin D1, cyclin D3, CDK2, and CDK4. Pretreatment with GW501516 significantly suppressed the PDGF-induced upregulation of those genes. The cell cycle inhibitor gene G0/G1 switch gene 2 (G0S2) has been proposed as a novel PPAR target gene [19]. The mRNA level of G0S2 in HPASMCs was significantly increased after GW501516 treatment. Moreover, pretreatment with GW501516 upregulated the expression of P27^kip1^ (P^27^), a CDK inhibitor that prevents cell proliferation by negatively regulating the activity of the cyclin-CDK complex. 

### 3.4. Ligand Activation of PPAR*δ* Inhibited PDGF-Induced Migration of HPASMCs

**Figure 4:**
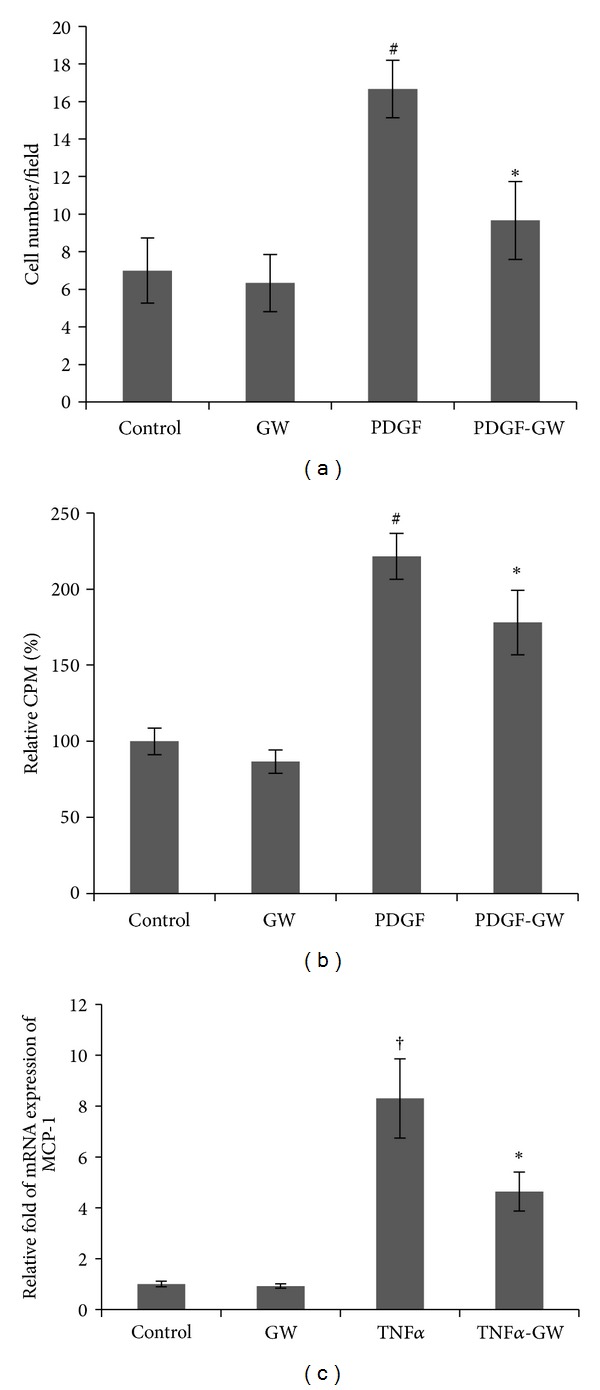
Regulation of migration, collagen synthesis, and MCP-1 expression in HPASMCs by GW501516. PDGF-stimulated (a) cell migration, (b) collagen synthesis, and (c) TNF-induced MCP-1 mRNA expression were significantly inhibited by GW501516. Experiments were performed in triplicate and repeated a minimum of three times. ^#^ *P* < 0.01, ^†^ *P* < 0.05  versus control; *P < 0.05 versus PDGF/TNF-treated cells.

To determine the effect of GW501516 on the migration of HPASMCs, we performed an in vitro transwell assay. As shown in Figure 4(a), an approximately 2-fold increase was observed in the cell migration of the PDGF-treated cells compared to controls. After 6 h of GW50151 pretreatment, the PDGF-induced migration of HPASMCs was significantly inhibited.

### 3.5. Ligand Activation of PPAR*δ* Inhibited Collagen Production by HPASMCs

To investigate the effect of PPAR*δ* on collagen synthesis, the ^3^H-proline incorporation was assessed. PPAR*δ* activation led to a significant decline in the ^3^H-proline incorporation induced by PDGF in HPASMCs, indicating a reduction in collagen synthesis (Figure 4(b)).

### 3.6. GW501516 Exerts an Anti-Inflammatory Effect in HPASMCs

The chemokine MCP-1 has been proposed to play an important role in the initiation and/or progression of PAH. Immunoreactivity for MCP-1 was detected in the endothelium, the smooth muscle cells, and the macrophages within the neointima in hypertensive large elastic pulmonary arteries. Anti-MCP-1 gene therapy attenuated PAH in rats [20]. We examined the anti-inflammatory effect of GW501516 on MCP-1 expression in HPASMCs treated with the proinflammatory factor TNF*α*. GW501516 significantly inhibited the TNF*α*-induced increase in MCP-1 mRNA expression (Figure 4(c)). 

## 4. Discussion 

In the present study, the expression of PPAR*δ* was found to be relatively high in HPASMCs compared with HPAECs. Moreover, three isoforms of PPARs were expressed in HPASMCs. In accordance with a previous finding that PPAR*δ* is abundantly expressed in VSMCs [18, 21], we confirmed that PPAR*δ* was the predominant isotype in HPASMCs. The vasculoproliferative disorders of PAH are characterized by the accumulation of vascular smooth muscle cells by proliferation and migration as well as extracellular matrix deposition. Cytokines and growth factors such as PDGF participate in these processes. We demonstrate that PPAR*δ* is abundantly expressed in HPASMCs and that PDGF upregulates PPAR*δ* expression in a time- and dose-dependent manner in HPASMCs; this finding is consistent with a previous report of PDGF inducing PGI2 expression in vascular cells [22]. These further suggest that PPAR*δ* is involved in VSMCs proliferation during vascular lesion formation. This study provides evidence that the activation of PPAR*δ* by a specific ligand, GW501516, attenuates the proliferation and migration of HPASMCs as well as the collagen synthesis that occurs in response to PDGF. This finding is consistent with a previous study reporting that PPAR*δ* is upregulated in vascular smooth muscle cells during vascular lesion formation and that the upregulation of PPAR*δ* may be a vascular compensatory response [18, 23]. 

VSMCs proliferation is a major component of the vasculoproliferative disorders. Vascular injury results in the release of growth factors and cytokines that stimulate quiescent VSMCs to enter the cell cycle. Cell cycle progression is dependent on the expression and activation of specific enzymes CDKs, which form complexes with their regulatory subunits, the cyclins. The cyclin-CDK complexes formed in cell cycle progression are regulated by CDK inhibitors, such as p21/Cip1 and p27/Kip1 [24]. This study revealed that GW501516, a selective ligand of PPAR*δ*, diminished the proliferation of HPASMCs induced by PDGF. The expression of cell cycle regulatory genes in response to PPAR*δ* activation in HPASMCs was investigated. GW501516 reduced the PDGF-induced expression of CDK2, CDK4, cyclin D1, and cyclin D3. In agreement with these changes, the mRNA expression of p27 was increased by GW501516. In addition, the expression of the cell cycle inhibitory gene G0S2 was upregulated after GW501516 treatment. Gene G0S2 contains a functional PPRE in the promoter region and has been confirmed as a novel PPAR target gene [19, 25]. These further suggested that the ligand activation of PPAR*δ* is involved in cell proliferation through the modulation of cell cycle regulatory genes. These findings are consistent with a recent report that a PGI2 analog mediated PPAR*δ* activation and enhanced the transcriptional activation of the expression of p21/p27, which resulted in the antiproliferative effects in VSMCs [26]. However, considerable controversy remains concerning the role of PPAR*δ* in cell growth, specifically whether PPAR*δ* stimulates or inhibits cell proliferation. Some studies indicate that activating PPAR*δ* causes increased cell proliferation in several different types of cells, including endothelial cells, keratinocytes, and cancer cells [27–29]. Additionally, PPAR*δ* has been reported to play an antiapoptotic role resulting in cellular proliferation [30]. These discrepancies may be related to experimental variables, including the choice of cell and animal models, the particular background strain of PPAR*δ* null mice, and the sites of PPAR*δ* genetic knockouts. Further studies necessary to examine the specific mechanisms underlying the effects of the ligand activation of PPAR*δ* in HPASMCs should include additional dose response analyses and quantitative measures of cell proliferation as well as comparative analyses of PPAR*δ*-dependent changes in the expression of known PPAR*δ* target genes.

The results of this study showed that ligand activation of PPAR*δ* significantly attenuated the proliferation and migration of PHASMCs as well as the collagen synthesis that occurs in response to PDGF. The inhibitory effect of PPAR*δ* on PDGF-stimulated PHASMCs functions was modest. Further studies are required to investigate the integrated effects of PPAR*δ* on pulmonary vascular remodeling in vivo. The three PPARs have distinct but often complementary functions [31]. PPARs have been implicated in many normal and disease-related biological processes such as inflammation, tissue remodeling, and atherosclerosis. Studies show redundancy in the function of PPAR*α* and PPAR*δ* as transcriptional regulators of fatty acid homeostasis [31, 32]. It becomes clear that PPARs participate in the control of cell proliferation and differentiation. PPAR*α* inhibits VSMCs proliferation by blocking G1/S cell cycle transition, through the induction of the CDK inhibitor p16^INK4*α*^ [33]. PPAR*γ* ligands decrease VSMCs proliferation by inhibiting mitogen-induced degradation of the CDK inhibitors p21 and p27 [34]. PDGF promotes the expression of PPAR*γ* in VSMCs by a PI3-kinase/Akt signaling pathway [35]. It is not known whether an interaction between PPAR*δ* and PPAR*γ* exists. The overall effect of PPAR*δ* on pulmonary vascular remodeling should be further investigated both in vitro and in vivo.

PPARs are ligand-activated transcription factors. Once activated by a ligand, the receptors bind to the promoter elements of target genes. GW501516 is the first highly selective synthetic PPAR*δ* agonist available. GW501516 binds to PPAR*δ* with an IC50 of 1 nM and is at least 1000-fold more selective for PPAR*δ* compared with PPAR*α* and PPAR*γ* [36]. Growing evidence has demonstrated that the ligand activation of PPAR*δ* is involved in multiple biological process involving lipid metabolism, glucose homeostasis, cell differentiation, and inflammation. We now report that PPAR*δ* has a potent inhibitory effect on the PDGF stimulation of three major cell functions in HPASMCs, including proliferation, migration, and collagen synthesis. These data are consistent with the inhibitory effect of a PPAR*δ* agonist on keratinocyte proliferation and the ability of cardiac fibroblasts to synthesize collagen in response to angiotensin II [37, 38]. Additionally, the ligand activation of PPAR*δ* inhibited TNF-induced upregulation of MCP-1, which is consistent with its anti-inflammatory effects in vascular endothelial cells and in kidney [39, 40]. Taken together, our data demonstrate that the ligand activation of PPAR*δ* reduced the proliferation of HPASMCs, an effect that was associated with the regulation of cell cycle regulatory gene expression. Furthermore, PPAR*δ* activation reduced cell migration, collagen synthesis, and chemokine production. Because PPAR*δ* is able to inhibit multiple aspects of vascular remodeling, it may be a therapeutic target for slowing the progression of vascular remodeling in PAH.
